# Perivascular Adipose Tissue Inflammation and Coronary Artery Disease: An Autopsy Study Protocol

**DOI:** 10.2196/resprot.6340

**Published:** 2016-11-18

**Authors:** Daniela Souza Farias-Itao, Carlos Augusto Pasqualucci, Aline Nishizawa, Luiz Fernando Ferraz Silva, Fernanda Marinho Campos, Karen Cristina Souza da Silva, Renata Elaine Paraizo Leite, Lea Tenenholz Grinberg, Renata Eloah Lucena Ferretti-Rebustini, Wilson Jacob Filho, Claudia Kimie Suemoto

**Affiliations:** ^1^ Laboratory of Cardiovascular Pathology Department of Pathology – LIM22 University of Sao Paulo Medical School Sao Paulo Brazil; ^2^ Physiopathology in Aging Lab/Brazilian Aging Brain Study Group – LIM22 University of Sao Paulo Medical School Sao Paulo Brazil; ^3^ Department of Pathology University of Sao Paulo Medical School Sao Paulo Brazil; ^4^ Discipline of Geriatrics University of Sao Paulo Medical School Sao Paulo Brazil; ^5^ Memory and Aging Center Department of Neurology University of California San Francisco, CA United States; ^6^ Medical-Surgical Nursing Department University of Sao Paulo School of Nursing Sao Paulo Brazil

**Keywords:** coronary artery disease, atherosclerosis, inflammation, adipose tissue, macrophages, B lymphocytes, T lymphocytes

## Abstract

**Background:**

Perivascular adipose tissue (PAT) inflammation may have a role in coronary artery disease (CAD) pathophysiology. However, most evidence has come from samples obtained during surgical procedures that may imply in some limitations. Moreover, the role of B lymphocytes and inflammation in PAT that is adjacent to unstable atheroma plaques has not been investigated in humans using morphometric measurements.

**Objective:**

The objective of this study is to investigate the inflammation in PAT, subcutaneous, and perirenal adipose tissues (SAT and PrAT) among chronic CAD, acute CAD, and control groups in an autopsy study.

**Methods:**

Heart, SAT, and PrAT samples are collected from autopsied subjects in a general autopsy service, with the written informed consent of the next-of-kin (NOK). Sociodemographic and clinical data are obtained from a semistructure interview with the NOK. Coronary arteries are dissected and PAT are removed. Sections with the greatest arterial obstruction or unstable plaques, and the local with absence of atherosclerosis in all coronary arteries are sampled. PAT are represented adjacent to these fragments. Adipose tissues are fixed in 4% buffered paraformaldehyde solution and analyzed immunohistochemically for macrophages (CD68), macrophage polarization (CD11c for proinflammatory and CD206 for anti-inflammatory), B lymphocytes (CD20), and T lymphocytes (CD3). Slides will be scanned, and inflammatory cells will be quantified in 20 random fields. Participants will be categorized in CAD groups, after morphometric measurement of arterial obstruction and plaque composition analysis in accordance with American Heart Association classification. Three study groups will be investigated: acute CAD (at least one unstable plaque); chronic CAD (≥50% arterial obstruction); and controls (<50% arterial obstruction). Inflammatory cells in PAT, SAT, and PrAT will be counted and compared between groups using multivariate linear regression, adjusted for age, body mass index, hypertension, diabetes, alcohol use, and smoking.

**Results:**

We present the methods of our study that was developed from 2 pilots. Currently, data collection and tissue processing are ongoing. Data collection, histology and immunochemistry procedures, and quantification of all inflammatory cells are expected to be concluded within 1 year.

**Conclusions:**

This study will contribute for the understanding of the mechanisms of CAD pathophysiology because it will help to clarify the role of inflammation both in chronic and acute CAD.

## Introduction

Cardiovascular disease is the leading global cause of death, accounting for 17.3 million deaths per year worldwide, and its prevalence is expected to grow to 23.6 million by 2030 [1,2]. Moreover, ischemic heart disease was the most frequent cause of disability-adjusted life years in 2012 [3]. Atherosclerosis is the main cause of cardiovascular disease deaths [4], and it is a chronic disease that can evolve into acute events related to vasospasm, thrombosis of advanced plaques, and embolism. The progression of atherosclerosis may be accelerated by inflammation [5]. Inflammation of the epicardial adipose tissue (EAT) has been linked to coronary artery disease (CAD) pathophysiology. The EAT has been reported to show high levels of inflammatory cytokines [6] and infiltration of leukocytes [7], particularly macrophages and T lymphocytes [6,8]. These changes appear to reflect a chronic proinflammatory response that is mediated by polarized macrophages [9] and is restricted to the heart [6,8,9].

Perivascular adipose tissue (PAT) surrounds most systemic blood vessels, except for the cerebral circulation; this may be a specialized type of adipose tissue related to inflammation and CAD severity [10]. PAT adipocytes have been found in the lamina adventitia of coronary arteries and the aorta [11,12]. Inflammatory cells, such as macrophages and T lymphocytes, were found between the PAT and the aorta adventitia [11]. PAT thickness has been associated with coronary artery calcification, cardiovascular risk factors [13], CAD burden [14], and the degree of atheroma plaque stenosis [15]. The number of macrophages in PAT has been related to the size and characteristics of the atheroma plaque (lipid core, calcification, collagen, and smooth muscle cell content), and to the degree of plaque infiltration by macrophages and lymphocytes. However, some limitations of previous studies should be considered. First, the location of PAT was not defined by its proximity to the atheroma plaque, but by its distance from the coronary artery ostium [15]. Moreover, the dissection of EAT was not conducted adjacent to the most relevant atherosclerotic plaque (periplaque PAT) in most studies, and no comparison was performed with a control area far from the atheroma plaque in the same individual. Second, the association between PAT inflammation and acute CAD was only investigated by imaging studies and no autopsy studies have been conducted to corroborate this association [16]. Third, although previous evidence suggested an association between PAT inflammation and CAD, most studies were conducted using samples collected during surgical procedures, which could had initiated the inflammatory process; the observed changes may therefore be unrelated to the chronic inflammation associated with atherosclerosis [17]. Finally, infiltration of B lymphocytes in PAT has not been investigated using autopsy studies.

The present manuscript aimed to describe standardized methods that were developed to investigate the association between inflammation of PAT and CAD in an autopsy study. The specifics aims of our study are to (1) investigate the association of macrophages, T and B lymphocytes with chronic CAD, acute CAD, and controls, (2) investigate the correlation between number of inflammatory cells in periplaque PAT and percentage of arterial obstruction, (3) investigate the association between number of inflammatory cells in periplaque PAT and atheroma plaque composition, and (4) compare the number of macrophages, polarized macrophages, T and B lymphocytes in periplaque PAT with the number of the same inflammatory cells in a control area far from the atheroma plaque, in SAT and in PrAT in the same individual.

## Methods

### Study Design and Setting

This observational cross-sectional autopsy study was approved by the local ethics committee. Written informed consent is obtained from the next-of-kin (NOK) of all participants before starting any study procedure.

The Sao Paulo Autopsy Service (SPAS) is a general autopsy service based at the University of Sao Paulo that performs approximately 13,000 autopsies per year. In Sao Paulo city, autopsy is mandatory when an individual dies from a natural cause, and where the cause of death is unclear or the individual did not have medical assistance. Since 2004, the Brazilian Aging Brain Study Group has sourced autopsy material from SPAS to investigate the normal and pathological aging brain [18]. The present study employed the same approach to collect adipose tissue samples and hearts.

### Recruitment

**Figure 1:**
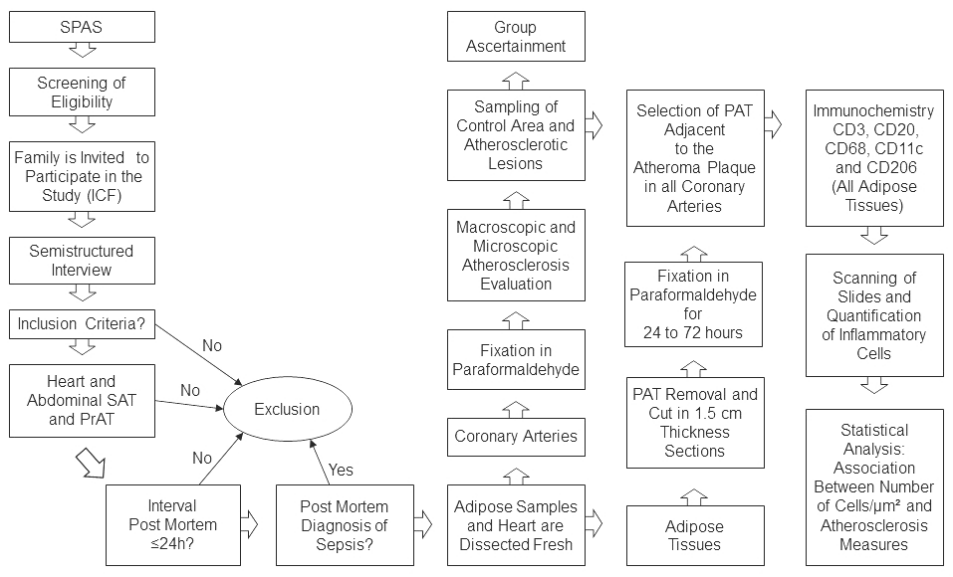
Study outline. SPAS: Sao Paulo Autopsy Service; ICF: informed consent form; SAT: subcutaneous adipose tissue; PrAT: perirenal adipose tissue; PAT: perivascular adipose tissue.

While the NOK waits for the autopsy of their deceased family member, they are invited to participate in this study. Nurses explain the aim of the study to the NOK and ask for their signature on an informed consent form. For all subjects to be included in this study, the NOK needs to have at least weekly interactions with the deceased and can therefore provide reliable information. A semistructured interview relating to sociodemographic and clinical information is conducted with each NOK prior to the collection of tissue samples (Figure 1).

### Study Population

The inclusion criteria for this study are: individuals of age ≥30 years at the time of death; a post mortem interval of ≤24 hours; a NOK with at least weekly contact with the deceased in the 6 months prior to death; and the availability of heart, SAT, and PrAT tissues from the medical pathologist. We included participants aged 30 years or older because advanced atherosclerotic plaques could be found in individuals with at least 30-years old at death [19].

The exclusion criteria employed in this study include: inconsistent clinical data provided by the NOK; adipose tissue fixation >72 hours; the use of corticosteroids or immunosuppressants, the presence of autoimmune disease, pericarditis, pericardial effusion, myocarditis, endocarditis, Chagas’ disease, hemopericardium, cardiac tamponade, coronary artery stent, or previous cardiac surgery. Patients with these conditions are excluded because they could induce or suppress inflammation in an atheroma plaque-independent manner. During the autopsy, samples of the major organs (heart, lung, spleen, kidney, and liver) are collected, processed, and stained with hematoxylin and eosin. Individuals that have an infection and show 2 or more criteria associated with sepsis in these samples, as defined previously [20,21], are also excluded from this study.

### Clinical Evaluation

The cause of death is determined by certified pathologists, based on the autopsy. In addition to the semistructured interview relating to the deceased’s sociodemographic information (including age, race, and education in years completed) [19,22]. The NOK provide clinical information relating to diagnosis (hypertension, diabetes, CAD, heart failure, dyslipidemia, or stroke) and lifestyle (physical activity, alcohol use, and smoking). Body mass index is calculated using the weight and height measured in a supine position, while the deceased have no clothes or shoes before the autopsy exam.

### Tissue Sampling

The heart is collected to obtain PAT and coronary arteries. SAT is removed from the region of the umbilical scar and PrAT from the kidney. The heart, SAT, and PrAT is washed in running water to remove blood and clots.

### Pilot Studies for Tissue Fixation

**Figure 2:**
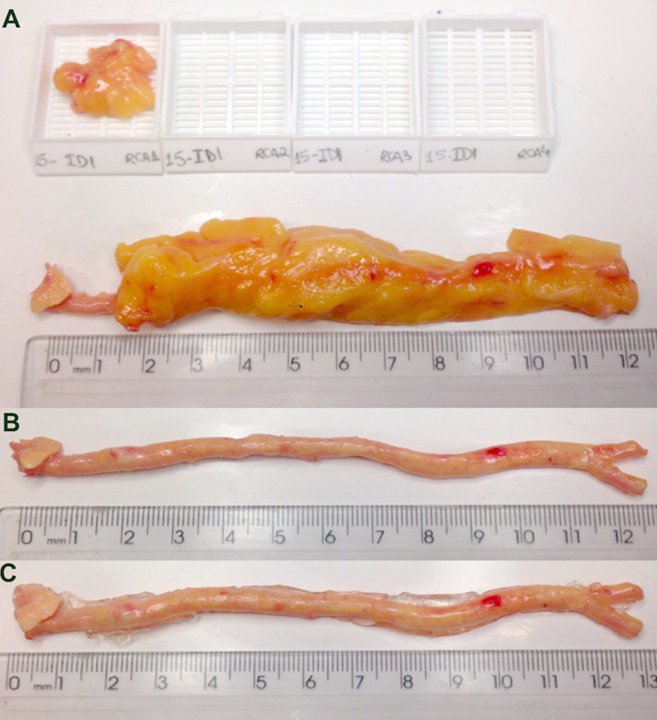
Perivascular adipose tissue (PAT) and coronary artery preparation of pilot 2. A: Agar was initially injected in the ostium of right coronary artery, the heart was cooled, and PAT was sampled at 1.5 cm intervals from the ostium until the final trajectory of the coronary artery. B: After PAT removal, the coronary artery was flat partially. C: The revised fixation procedure included at least two agar injections, and the coronary arteries were effectively open.

**Figure 3:**
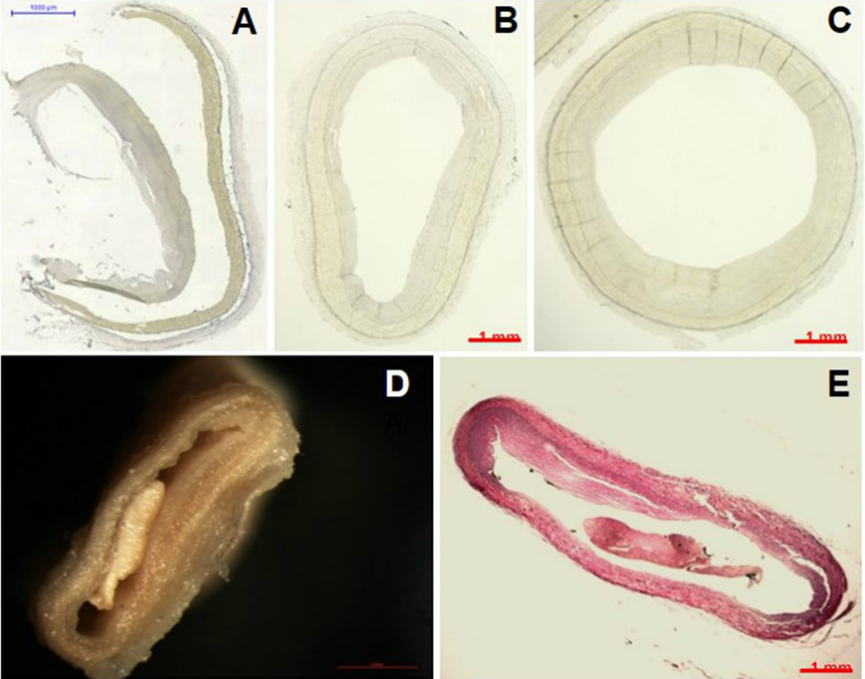
Fixation of the coronary artery. A: Coronary artery without agar injection and fixation in 10% formaldehyde solution. B: Coronary artery with agar injection and fixation in 10% formaldehyde solution. C: Coronary artery with agar injection and fixation in 4% buffered paraformaldehyde solution. D: Coronary artery flattening observed following heart fixation by immersion in alcohol prior to dissection of the coronary arteries (photographed macroscopically). E: Section of the same tissue shown in panel D, photographed microscopically after staining.

Two processes were compared for heart fixation. The first involved fixation of the heart (with EAT) by immersion in 70% alcohol for at least 24 hours. After this period, we dissected the right coronary artery, left coronary artery trunk, anterior descending coronary artery, and circumflex coronary artery, together with 1 cm of the adjacent PAT and all EAT was removed from myocardium. PAT was removed systematically and cut into 1.5 cm sections, starting from the ostium (Figure 2). After these dissections, PAT and EAT were weighted using an electronic scale. Subsequently, agar was injected into the lumen of each coronary artery, which was then stored in 10% formaldehyde solution for 5 days. However, we observed flattening of the coronary arteries (Figures 3 D, 3 E), even with agar injection.

Therefore, we changed our fixation protocol. The second fixation process involved the coronary artery dissection with the adjacent PAT immediately after heart collection to test whether the dissection of fresh coronary arteries and PAT would be more effective. The heart was incubated at −20°C for 2 minutes prior to injecting agar inside each coronary artery, and then the heart was placed at −20°C for another 2 minutes to solidify the agar. The coronary arteries were then dissected as described above and PAT was removed. The second fixation protocol has two advantages. First, the injection of agar before artery dissection allows agar infiltration until minor branches. Second, when we dissect the coronary arteries, there are already solidified agar inside the arterial lumen, facilitating arterial opening after repeated injections of agar (Figures 2 B, 2 C). The problem of injecting agar only after heart fixation is that coronary arteries are already hardened by alcohol. Moreover, when we injected agar after coronary dissection, most of the agar did not stay inside the lumen, preventing arterial opening (Figures 3 D, 3 E).

We also investigated whether the 10% formaldehyde solution and 4% buffered paraformaldehyde solution (pH 7.2‑7.4) influenced coronary artery fixation. Coronary arteries samples were identified and then fixed for 5 days. We used the same fragment of coronary artery divided in 3 to compare agar injection procedures and fixatives. Agar prevented artery flattening (Figures 3 B, 3 C), which could interfere with the measurement of arterial stenosis. Moreover, the presence of the agar protected the intima layer from 10% formaldehyde solution damage (Figure 3 A, 3 B). In addition, the 4% buffered paraformaldehyde solution was less aggressive to the tissue than 10% unbuffered formaldehyde solution (Figure 3 C). Therefore, we chose to dissect fresh coronary arteries and PAT and used 4% buffered paraformaldehyde solution as the fixative.

**Figure 4:**
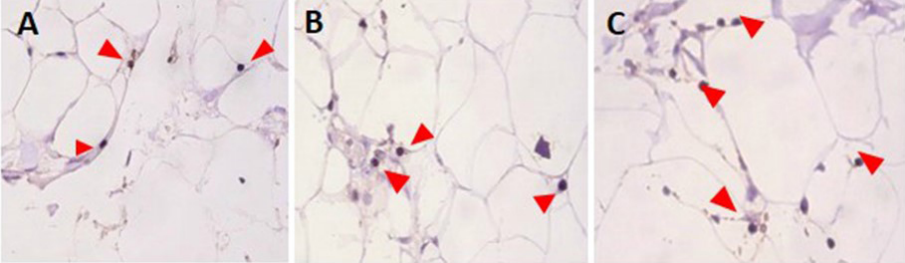
Immunochemistry of perivascular adipose tissue (PAT) using an anti-CD3 primary antibody. PAT was fixed for A: 24 hours; B: 48 hours; and C: 72 hours. Red arrows indicate CD3-positive T lymphocytes. All images were obtained using a microscope at 20× magnification.

We compared adipose tissue fixation times of 24, 48, and 72 hours in 4% buffered paraformaldehyde solution. Fixation times >72 hours were not tested because they can lead to a loss of antigenicity or the production of formic acid, which can impair identification of immune cells [23,24]. All primary antibodies were tested in positive control tissue sections (lung, tonsil, and lymph node) to facilitate standardization (Table 1). No difference in inflammatory cell staining was found using the 3 different fixation times (Figure 4). Thus, although 24 hours was chosen as the fixation time for subsequent analyses, this could be extended for up to 72 hours if necessary.

**Table 1:** Primary antibodies used to identify inflammatory cells.

Target	Specification	Dilution	Inflammatory cell
CD3	Polyclonal rabbit anti-human	1:1500	T lymphocytes [6]
CD20	Monoclonal mouse anti-human clone L26	1:12,000	B lymphocytes
CD68	Monoclonal mouse anti-human clone KP-1	1:5000	Macrophages [6,8,15]
CD11c	Monoclonal rabbit anti-human clone EP1347Y	1:400	Macrophages polarized M1 [9]
CD206	Monoclonal mouse anti-human clone 5C11	1:1500	Macrophages polarized M2 [9]

**Figure 5:**
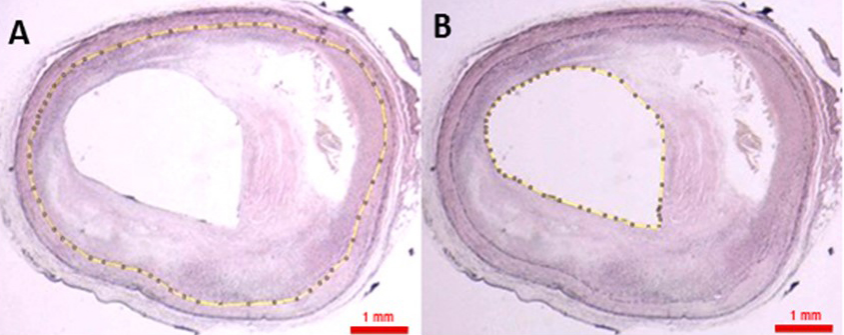
Area measurements: the internal elastic lamina (IEL) and lumen. A: Area delineated by the IEL. B: Area of the lumen.

### Evaluation of Coronary Artery Atherosclerosis

#### Macroscopic Evaluation

After fixation, coronary arteries are washed in running water for 30 minutes to remove excess paraformaldehyde solution. All coronary arteries are cut in 5-mm sections to identify the section with the largest obstruction or unstable plaques (eg, hemorrhage or thrombus), using a magnifying glass (Emporionet LP 500). An area without atherosclerosis that is distal to the section with the largest degree of arterial stenosis is also sampled. The number of atherosclerotic plaques is counted along the artery, as a measure of the extent of atherosclerosis. The agar is removed from inside the arterial lumen and the sections are photographed using a stereomicroscope (Nikon SMZ 1000).

#### Microscopic Evaluation

The coronary arteries are decalcified, dehydrated, diaphonized, and immersed in paraffin prior to cutting 4 μm sections, using a microtome and staining with Verhoeff’s stain, as well as hematoxylin and eosin. The sections are then photographed using a stereomicroscope. The percentage of arterial obstruction is measured by morphometric methods using an image processing software (ImageJ). We measure the area of the lumen and the area delineated by the internal elastic lamina (IEL) (Figure 5). To calculate the percentage of arterial obstruction, we divide the difference between the area within the IEL and the area of the lumen by the area within the IEL, and multiplied the result by 100 [25]. Moreover, we classify the atheroma plaque in accordance with the American Heart Association (AHA) criteria [19].

### CAD Classification

After we complete the tissue processing, participants will be classified into 3 groups: chronic CAD, with ≥50% obstruction in at least one artery [7], and an AHA classification different than VI [25]; acute CAD, with at least one atheroma plaque with an AHA classification of VI (unstable plaque) [19]; with no meaningful CAD [7], and an AHA classification different than VI [19]. If the sample size allows, we will perform a subanalysis with participants without arterial obstruction as the control group.

### Histological and Immunochemical Procedures in Adipose Tissues

PAT, SAT, and PrAT are cut into 4 µm sections, applied to silanized slides (3-aminopropyltriethoxysilane), and immersed in paraffin. Prior to immunochemistry, the sections of PAT that correspond with sampled coronary artery fragments, SAT, and PrAT are deparaffinized by placing the slides in hot xylene in an oven at 60°C to 65°C for 5 minutes and then dipping in 3 baths of cold xylene. The sections are then hydrated in 95% alcohol, followed by 70% alcohol, washed in tap water and deionized water, and placed in phosphate buffer, pH 7.4. Antigen recovery is performed in 10 mM citric acid, pH 6, at a high temperature in a pressure cooker. Endogenous peroxidase is blocked using 3% hydrogen peroxide prior to incubating the slides with the indicated primary antibodies in the presence of 1% bovine serum albumin for 24 hours at 4°C. The slides are then incubated with the appropriate horseradish peroxidase-conjugated secondary antibody and EasyLink One and the signal is generated using the chromogen, diaminobenzidine. The sections are counterstained using Harris hematoxylin.

### Inflammatory Cell Counting

The slides are scanned and analyzed at 40× magnification using the Pannoramic Viewer software. This program hides the slide identification so that the operator is blinded to the subject’s diagnosis, the source and type of adipose tissue, and hotspots (ie, accumulation of inflammatory cells). Twenty random fields with 600 µm of diameter were also analyzed with no magnification (cells can only be observed with precision at 20× magnification). This process is systematic and large cell agglomerates (identified in >20× magnification) are avoided. Inflammatory cells are counted per the primary antibody staining and these results were expressed as the number of cells per micrometers squared.

### Statistical Analyses

To calculate the sample size, we used a previous study that found an effect size of 0.93, with a mean standard deviation of 44 (SD 21) inflammatory cells/µm² in a CAD group and 24 (SD 22) inflammatory cells/µm² in a control group [7]. Assuming an alpha of 5% and a power of 90%, we estimated that 26 subjects would be needed in each group, giving a total sample size of 78. The independent variable of our study is CAD and the dependent variables are the numbers of macrophages, polarized macrophages, and lymphocytes (B and T) in PAT, PrAT, and SAT. The groups will be compared regarding demographic and clinical variables, using chi-square test for categorical variables and one-way analysis of the variance (ANOVA) for continuous ones. The weight of EAT will be compared among groups using one-way ANOVA. A multivariate linear regression model will be used to compare the dependent and independent variables, adjusted for age, hypertension, diabetes mellitus, body mass index, alcohol use, and smoking. The significance level for all tests will be set at 5% in two-tailed tests. We will use STATA 13.0 to perform these analyses.

## Results

Currently, data collection and tissue processing are ongoing. The data collection, histology and immunochemistry procedures, and quantification of inflammatory cells are expected to be concluded by May 2017.

## Discussion

### Clinical Implications

Although EAT thickness and volume can be evaluated using imaging methods [26], the number and type of inflammatory cells in PAT can only be determined by pathological examination. In addition, autopsy studies can employ morphometric methods to calculate the degree of plaque stenosis and plaque composition. Finally, they can exclude other inflammatory diseases, which could bias the results. Here, we describe a protocol for sample processing, immunochemical analyses, and morphometric measurements of coronary artery stenosis and inflammatory cells in adipose tissues.

Although the association of CAD with macrophages and T lymphocytes has been investigated previously [6,8,9,15], a study of this association using a range of controls, including the analysis of SAT, PrAT, and EAT distal from the atheroma plaque will help to determine the extent of the inflammatory process. Moreover, the contribution of B lymphocytes in PAT to the atherosclerotic process has not yet been investigated, and the association between EAT inflammation and acute CAD has only been investigated using imaging methods, thus precluding the direct quantification of inflammatory cells [6].

It is possible that inflammation in PAT could contribute locally to the development of the atherosclerotic plaque, as suggested by previous imaging [7,6] and autopsy studies [15]. The mechanism underlying these findings is not yet established, but it is biologically plausible. Infiltration of adipocytes in the PAT was found in the adventitial layer, which could have direct influence on the inflammation in coronary arteries [12,27]. In addition, the vasa vasorum, which is in close contact with the PAT [10,26], grows in the direction of the intimal layer [28] when intima media thickness is present. The infiltration of inflammatory cells in adventitial layer may contribute to this angiogenesis.

PAT inflammation may be a measurable and modifiable risk factor that could be used in clinical practice. Some studies have investigated interventions that aim to reduce the inflammatory burden, for example by reducing the EAT volume via a reduction of the total body weight or by promoting the conversion of white adipose tissue into brown adipose tissue, which is associated with a decreased risk of obesity-related disorders. Other studies have investigated drugs that modulate immune receptors to reduce inflammation [29,30].

However, while inflammation and PAT thickness have been suggested to show positive associations with the degree of arterial stenosis [31], a paradox has been observed in clinical practice, whereby a low EAT volume was associated with a reduced myocardial salvage area and a larger infarct size in patients with a first ST-segment elevation myocardial infarction [32]. Therefore, studies of the roles of different inflammatory cells in PAT are important to elucidate CAD pathophysiology and identify new therapeutic targets.

### Strengths and Limitations

Nevertheless, one limitation of the present study is that the sociodemographic and clinical information are collected after death from the NOK. To improve the accuracy of this information, we only include NOK with daily or weekly interactions with the deceased. Moreover, the reliability of the post mortem interview had been demonstrated by a previous study from our group, which showed a high sensitivity (87%) and specificity (94%) using this approach [33]. In addition, the main variables analyzed in the present study (atherosclerotic burden and inflammatory cell numbers) are measured objectively.

Here, we described the protocol that we are using to investigate the association between CAD and inflammation in adipose tissues. Particularly, we described in details the pilot studies that we performed to fixate and process the arteries and adipose tissues. These measures are important to allow for unbiased morphometric measures of atherosclerosis and inflammatory cell counting.

## Abbreviations

AHA: American Heart Association
ANOVA: analysis of the variance
CAD: coronary artery disease
CAPES: Coordenação de Aperfeiçoamento de Pessoal de Nível Superior
EAT: epicardial adipose tissue
FAPESP: Fundação de Amparo à Pesquisa do Estado de São Paulo
ICF: informed consent
IEL: internal elastic lamina
NOK: next-of-kin
PAT: perivascular adipose tissue
PrAT: perirenal adipose tissue
SAT: subcutaneous adipose tissue
SPAS: Sao Paulo Autopsy Service

## References

[1] Mozaffarian D, Benjamin EJ, Go AS, Arnett DK, Blaha MJ, Cushman M, de Ferranti S, Després JP, Fullerton HJ, Howard VJ, Huffman MD, Judd SE, Kissela BM, Lackland DT, Lichtman JH, Lisabeth LD, Liu S, Mackey RH, Matchar DB, McGuire DK, Mohler ER, Moy CS, Muntner P, Mussolino ME, Nasir K, Neumar RW, Nichol G, Palaniappan L, Pandey DK, Reeves MJ, Rodriguez CJ, Sorlie PD, Stein J, Towfighi A, Turan TN, Virani SS, Willey JZ, Woo D, Yeh RW, Turner MB, American Heart Association Statistics Committee and Stroke Statistics Subcommittee (2015). Heart disease and stroke statistics - 2015 update: a report from the American Heart Association. Circulation.

[2] World Health Organization (2013). Projections of mortality and causes of death, 2015 and 2030.

[3] World Health Organization (2014). Disease burden: estimates for 2000-2012.

[4] Herrington W, Lacey B, Sherliker P, Armitage J, Lewington S (2016). Epidemiology of atherosclerosis and the potential to reduce the global burden of atherothrombotic disease. Circ Res.

[5] Ketelhuth DFJ, Hansson GK (2016). Adaptive response of T and B cells in atherosclerosis. Circ Res.

[6] Mazurek T, Zhang L, Zalewski A, Mannion JD, Diehl JT, Arafat H, Sarov-Blat L, O'Brien S, Keiper EA, Johnson AG, Martin J, Goldstein BJ, Shi Y (2003). Human epicardial adipose tissue is a source of inflammatory mediators. Circulation.

[7] Konishi M, Sugiyama S, Sato Y, Oshima S, Sugamura K, Nozaki T, Ohba K, Matsubara J, Sumida H, Nagayoshi Y, Sakamoto K, Utsunomiya D, Awai K, Jinnouchi H, Matsuzawa Y, Yamashita Y, Asada Y, Kimura K, Umemura S, Ogawa H (2010). Pericardial fat inflammation correlates with coronary artery disease. Atherosclerosis.

[8] Hirata Y, Kurobe H, Akaike M, Chikugo F, Hori T, Bando Y, Nishio C, Higashida M, Nakaya Y, Kitagawa T, Sata M (2011). Enhanced inflammation in epicardial fat in patients with coronary artery disease. Int Heart J.

[9] Hirata Y, Tabata M, Kurobe H, Motoki T, Akaike M, Nishio C, Higashida M, Mikasa H, Nakaya Y, Takanashi S, Igarashi T, Kitagawa T, Sata M (2011). Coronary atherosclerosis is associated with macrophage polarization in epicardial adipose tissue. J Am Coll Cardiol.

[10] Szasz T, Webb RC (2012). Perivascular adipose tissue: more than just structural support. Clin Sci.

[11] Henrichot E, Juge-Aubry CE, Pernin A, Pache JC, Velebit V, Dayer JM, Meda P, Chizzolini C, Meier CA (2005). Production of chemokines by perivascular adipose tissue: a role in the pathogenesis of atherosclerosis?. Arterioscler Thromb Vasc Biol.

[12] Chatterjee TK, Stoll LL, Denning GM, Harrelson A, Blomkalns AL, Idelman G, Rothenberg FG, Neltner B, Romig-Martin SA, Dickson EW, Rudich S, Weintraub NL (2009). Proinflammatory phenotype of perivascular adipocytes: influence of high-fat feeding. Circu Res.

[13] de Vos AM, Prokop M, Roos CJ, Meijs MFL, van der Schouw YT, Rutten A, Gorter PM, Cramer MJ, Doevendans PA, Rensing BJ, Bartelink ML, Velthuis BK, Mosterd A, Bots ML (2007). Peri-coronary epicardial adipose tissue is related to cardiovascular risk factors and coronary artery calcification in post-menopausal women. Euro Heart J.

[14] Iacobellis G, Willens HJ (2009). Echocardiographic epicardial fat: a review of research and clinical applications. J Am Soc Echocardiogr.

[15] Verhagen SN, Vink A, van der Graaf Y, Visseren FLJ (2012). Coronary perivascular adipose tissue characteristics are related to atherosclerotic plaque size and composition: a post-mortem study. Atherosclerosis.

[16] Mazurek T, Kochman J, Kobylecka M, Wilimski R, Filipiak KJ, Królicki L, Opolski G (2014). Inflammatory activity of pericoronary adipose tissue may affect plaque composition in patients with acute coronary syndrome without persistent ST-segment elevation: preliminary results. Kardiol Pol.

[17] Dybdahl B, Wahba A, Lien E, Flo TH, Waage A, Qureshi N, Sellevold OFM, Espevik T, Sundan A (2002). Inflammatory response after open heart surgery: release of heat-shock protein 70 and signaling through toll-like receptor-4. Circulation.

[18] Grinberg LT, Ferretti REL, Farfel JM, Leite REP, Pasqualucci CA, Rosemberg S, Nitrini R, Saldiva PHN, Jacob-Filho W, Brazilian Aging Brain Study Group (2007). Brain bank of the Brazilian aging brain study group—a milestone reached and more than 1,600 collected brains. Cell Tissue Bank.

[19] Stary HC (2000). Natural history and histological classification of atherosclerotic lesions: an update. Arterioscler Thromb Vasc Biol.

[20] Lucas S (2007). The autopsy pathology of sepsis-related death. Curr Diagn Pathol.

[21] Tsokos M (2007). Postmortem diagnosis of sepsis. Forensic Sci Int.

[22] Ferretti REL, Grinberg LT, Leite REP, Farfel JM, Pasqualucci CA, Nitrini R, Jacob-Filho W (2009). Bank of human encephala: an important tool for the study of brain aging. O Mundo da Saúde.

[23] Syrbu SI, Cohen MB (2011). An enhanced antigen-retrieval protocol for immunohistochemical staining of formalin-fixed, paraffin-embedded tissues. Methods Mol Biol.

[24] Fox CH, Johnson FB, Whiting J, Roller PP (1985). Formaldehyde fixation. J Histochem Cytochem.

[25] Suemoto CK, Nitrini R, Grinberg LT, Ferretti REL, Farfel JM, Leite REP, Menezes PR, Fregni F, Jacob-Filho W, Pasqualucci CA, Brazilian Aging Brain Study Group (2011). Atherosclerosis and dementia: a cross-sectional study with pathological analysis of the carotid arteries. Stroke.

[26] Rajsheker S, Manka D, Blomkalns AL, Chatterjee TK, Stoll LL, Weintraub NL (2010). Crosstalk between perivascular adipose tissue and blood vessels. Curr Opin Pharmacol.

[27] Tavora F, Kutys R, Li L, Ripple M, Fowler D, Burke A (2014). Adventitial lymphocytic inflammation in human coronary arteries with intimal atherosclerosis. Cardiovasc Pathol.

[28] Williams JK, Heistad DD (1996). Structure and function of vasa vasorum. Trends Cardiovasc Med.

[29] Iacobellis G (2016). Epicardial fat: a new cardiovascular therapeutic target. Curr Opin Pharmacol.

[30] Saely CH, Geiger K, Drexel H (2012). Brown versus white adipose tissue: a mini-review. Gerontology.

[31] Mazurek T, Kobylecka M, Zielenkiewicz M, Kurek A, Kochman J, Filipiak KJ, Mazurek K, Huczek Z, Królicki L, Opolski G (2016). PET/CT evaluation of (18)F-FDG uptake in pericoronary adipose tissue in patients with stable coronary artery disease: Independent predictor of atherosclerotic lesions’ formation?. J Nucl Cardiol.

[32] Gohbara M, Iwahashi N, Akiyama E, Maejima N, Tsukahara K, Hibi K, Kosuge M, Ebina T, Umemura S, Kimura K (2016). Association between epicardial adipose tissue volume and myocardial salvage in patients with a first ST-segment elevation myocardial infarction: an epicardial adipose tissue paradox. J Cardiol.

[33] Ferretti REL, Damin AE, Brucki SMD, Morillo LS, Perroco TR, Campora F, Moreira EG, Balbino ES, Lima MCA, Battela C, Ruiz L, Grinberg LT, Farfel JM, Leite REP, Suemoto CK, Pasqualucci CA, Rosemberg S, Saldiva PHN, Jacob-Filho W, Nitrini R, Brazilian Aging Brain Study Group (2010). Post-mortem diagnosis of dementia by informant interview. Dement Neuropsychol.

